# Dosimetric benefits of gantry‐static couch‐motion (GsCM) technique for breast boost radiation therapy: Reduced dose to organs‐at‐risk and improved dosimetric indices

**DOI:** 10.1002/acm2.12969

**Published:** 2020-07-05

**Authors:** Gurtej S. Gill, Raphael Y. Jakubovic, Jameson Baker, Terry Button, Jenghwa Chang

**Affiliations:** ^1^ Department of Radiation Medicine Center for Advanced Medicine Northwell Health New York NY USA; ^2^ Department of Biomedical Engineering Stony Brook University New York NY USA; ^3^ Department of Radiation Medicine Hamilton Health Services Walker Family Cancer Center Ontario CA USA; ^4^ Department of Radiology and Biomedical Engineering Stony Brook University New York NY USA

**Keywords:** 3D conformal external beam planning, breast boost, couch motion–based dose calculation, trajectory‐based delivery, wedge pair

## Abstract

To evaluate the clinical feasibility and dosimetric benefits of a novel gantry‐static couch‐motion (GsCM) technique for external beam photon boost treatment of lumpectomy cavity in patients with early‐stage breast cancer in comparison to three‐dimensional conformal radiotherapy (3D‐CRT), wedge pair in supine position (WPS), and wedge pair in decubitus position (WPD) techniques. A retrospective review was conducted on breast patients (right breast, n = 10 and left breast, n = 10) who received 10 Gy boost after 50 Gy to whole breast. The treatment plans were generated using an isocentric‐based GsCM technique (a VMAT type planning approach) integrating couch rotational motion at static gantry positions. Static fields for each tangential side were merged using a Matlab^®^ script and delivered automatically within the Varian Truebeam**^TM^** STx in Developer Mode application as a VMAT arc (wide‐angular medial and short‐angular lateral arcs). The dosimetric accuracy of the plan delivery was evaluated by ion chamber array measurements in phantom. For both right and left breast boost GsCM, 3D‐CRT, WPS, and WPD all provided an adequate coverage to PTV. GsCM significantly reduced the ipsilateral lung V30% for right side (mean, 80%) and left side (mean, 70%). Heart V5% reduced by 90% (mean) for right and 80% (mean) for left side. Ipsilateral breast V50% and mean dose were comparable for all techniques but for GsCM, V100% reduced by 50% (mean) for right and left side. The automated delivery of both arcs was under 2 min as compared to delivering individual fields (30 ± 5 min). The gamma analysis using 2 mm distance to agreement (DTA) and 2% dose difference (DD) was 98 ± 1.5% for all 20 plans. The GsCM technique facilitates coronal plane dose delivery appropriate for deep‐seated breast boost cavities, with sufficient dose conformity of target volume paired with sparing of the OARs.

## 1 INTRODUCTION

Radiation therapy plays an important role for patients who undergo breast conservation therapy (BCT) which includes both breast conserving surgery (BCS) and radiotherapy. Breast conservation therapy preserves the breast normal tissue as much as possible without compromising survival. Breast conservation surgery as known as lumpectomy, quadrantectomy, partial mastectomy, or segmental mastectomy depending on how the tissue has been removed is important which includes resection of the primary tumor with or without axillary nodes followed by radiotherapy to eradicate the residual microscopic disease of the breast tissue.^1^, ^2^ According to cancer statistics^3^ in 2020, there were about 276 480 (30% of estimated new cases for all sites in female) new cases of breast cancer. In women, breast cancer has high incidence rate as compared to other types of cancer. Depending on the patient staging,^4^, ^5^ for early‐stage breast cancer with stage I and II, the conservative surgery and radiation therapy are standard alternatives to mastectomy. Radiation therapy after lumpectomy has been known for long‐term local control equivalent to mastectomy^5^, ^6^ on the order of 85–95% with similar survival outcomes. Furthermore, postlumpectomy radiation therapy is associated with reduction in local recurrence and improved overall survival rate as compared to surgery alone.^7^ Therefore, radiation therapy is considered a better approach for postlumpectomy treatment as compared to lumpectomy alone.

Patients who have early‐stage breast cancer and received a lumpectomy can get sequential boost (10–18 Gy) to postlumpectomy preceded by whole breast radiation therapy (46–50.4 Gy with 1.8 to 2 Gy daily fractions) as per the Radiation Therapy Oncology Group (RTOG) 1005.^5^ Current clinical practice is to treat the whole breast followed by a coned down boost to the lumpectomy cavity using electrons for superficial cavities and photons for deep‐seated cavities. While both photons and electrons aim for conformal irradiation to the target while minimizing the dose to organs‐at‐risk (OARs), selection of one versus the other should be carefully considered to avoid toxicity.^8^

In a previous publication,^9^ we have developed the clinical feasibility of gantry static couch motion (GsCM) technique for treating deep‐seated brain tumors. This technique was found beneficial for brainstem tumors or targets in the middle of optic chiasm and brainstem. The main advantageous of this approach in brain tumors was a sharp dose fall‐off anteriorly and posteriorly to the target which spared normal tissues such as optical track and brainstem. In the current study, two partial arcs were simulated by adding multiple conformal static fields for each side. The GsCM technique is conceptually similar to volumetric‐modulated arc therapy (VMAT) because it includes dose modulation. However, the modulation is achieved without inverse optimization. For GsCM the couch is dynamic with a static gantry (ie, GsCM utilizes a fixed gantry and rotating couch).

In this study we investigated the potential dosimetric advantages of the GsCM technique for sequential/concurrent boost of postlumpectomy cavities as compared to existing three‐dimensional conformal radiation therapy (3D‐CRT), conventional wedge pair in supine position (WPS), and wedge pair in decubitus position (WPD) techniques. The dose volume histogram (DVH) for lungs, normal breast tissue, and heart was calculated and compared with that of 3D‐CRT and conventional wedge pair (WPS, WPD) techniques. The dosimetric accuracy of the plan delivery was evaluated by ion chamber array measurements in phantom.

## 2 MATERIALS AND METHODS

In this study, the GsCM technique was implemented for the boost treatments of (n = 20) breast patients. This retrospective study has received an institutional review board (IRB#19‐1025) approval to conduct the comparison of different treatment planning techniques for breast boost treatments. This study is focused on women who have large breast size resulting in a postlumpectomy cavity that is >5 cm deep. Use of electrons in this case is inferior due to greater skin dose and inadequate coverage distally to the lumpectomy cavity. Therefore, comparison with electrons was excluded from this study.

### 2.A The gantry‐static couch‐motion (GsCM) technique

In this study the GsCM technique was implemented by first selecting the medial and lateral beams arranged with 2° delta couch angles. The GsCM concept begins somewhat similar to dynamic conformal arc, in the sense that each beam initially conforms to the planning target volume (PTV) plus margin at every segment, which is actually a fixed static beam. However, the MLCs were conformed with a static gantry and a variable couch. After, the dose calculation, the beam weighting was adjusted to achieve good conformity around the target. Next, a fluence editing option was used to make the dose distribution homogenous as possible within the PTV. The MLCs were then generated with a limit of 1 segment per field. The groups of static beams then became like VMAT with both dose rate and aperture modulation. The main differences between standard VMAT and our GsCM technique are the moving couch instead of gantry, use of nontraditional inverse optimization, and the dose delivery at 2° increments rather than continuous.

The simulated fields for GsCM technique were then transferred to MATLAB® for in‐house custom‐made script to combine all the modulated fields. This provides a single deliverable file in XML format. This XML file mirrors all the beam characteristics as compared to the planned treatment fields. Implementation was performed within the Truebeam® (Varian Medical Systems, Palo Alto, CA) developer mode application utilizing 6 MV energy. Varian developer mode application in the research environment allows for custom XML scripting by controlling all the mechanical axes of the LINAC including gantry, couch, collimator, jaws, and MLC control points for safe delivery on the LINAC.

### 2.B Patient selection, radiation dose prescription, and treatment volume

This retrospective study consisted of 20 patients previously treated with external beam photon radiation therapy. All patients previously received 50 Gy in 25 fractions to whole right or left breast. In each case a photon boost of 10 Gy in 4 fractions was also delivered to the lumpectomy cavity. These 20 patients [(left breast (n = 10) and right breast (n = 10)] were replanned for 3D‐CRT, WPS, WPD, and for GsCM techniques for dosimetric comparison. This study focused on the “ARM I” criteria of RTOG 1005 protocol as a reference for inclusion of early‐stage breast cancer patients with stage I and II eligible for receiving radiation therapy. Random selection of these patients (treated from July 2019 to December 2019) with tumors located in the central, upper‐inner, upper‐outer, lower‐inner, and lower‐outer quadrants was included for this study. The number of patients, diagnosis, location of tumor, and their respective volumes are shown in Table 1.

**Table 1:** Primary tumor laterality and patient characteristics.

Total patients	Side of lesion	Stage	Quadrant	Excision cavity volume (cm^3^), mean ± SD	PTV volume (cm^3^), mean ± SD	Whole breast volume (cm^nn^), mean ± SD
10	Right breast	I and II	Lower inner, Upper outer, Central, Upper outer, and Upper inner	8.10 ± 1.80	100.29 ± 18.85	1594.65 ± 553.95
10	Left breast	I and II	Lower inner, Central, Upper inner, Lower outer, and Upper outer	14.01 ± 3.32	105.15 ± 9.29	1489.32 ± 393.22

**Fig. 1:**
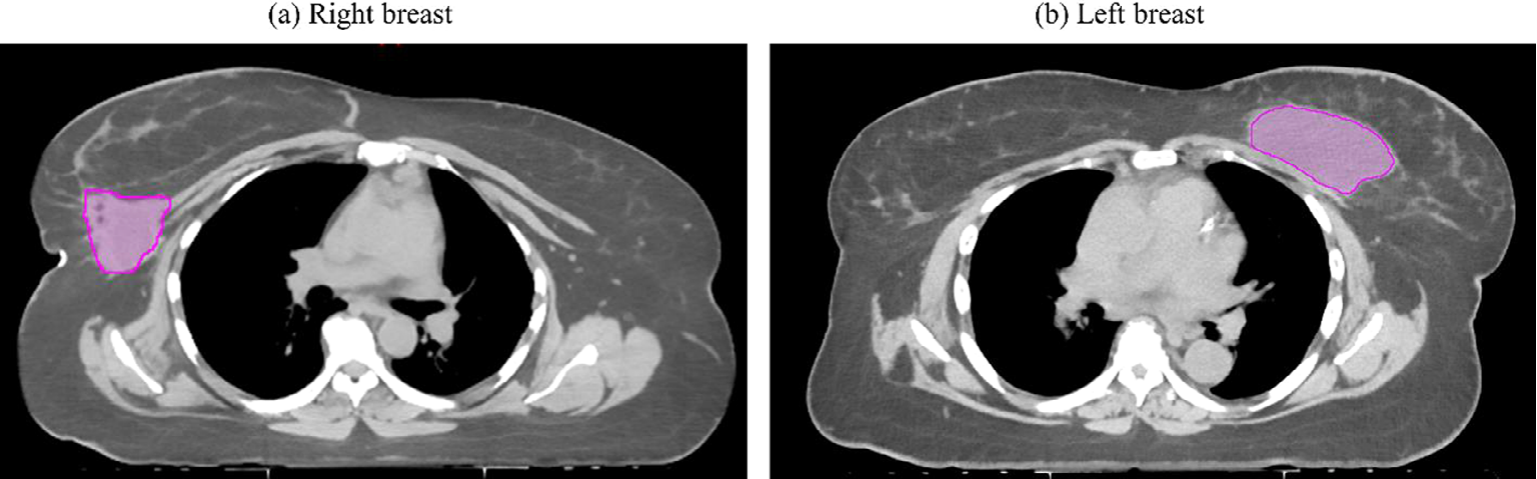
The location of lumpectomy cavity is shown for a right breast (a) and a left breast (b) patient included in this study. In both pictures, the lumpectomy cavities were contoured in pink color as the gross tumor volume.

All patients were scanned under CT simulation (Philips medical systems, Netherlands) in house (2 mm slice thickness) in a supine position for the primary whole breast and in the decubitus position for the coned‐down lumpectomy boost. The CT scan in the decubitus position was acquired for all patients under study since the cavity is deep seated and not easy to target in the supine position. The gross tumor volume (GTV) was contoured by the radiation oncologist on both supine and decubitus CT studies which is the standard of practice at our center. The lumpectomy cavity plus a 1.0 cm expansion was applied to generate the clinical target volume (CTV) on both supine CT and decubitus CT. The planning target volume (PTV) was generated by adding 0.5 cm margin to CTV (a total of 1.5cm margin around the GTV) to account for set up uncertainty. Figure 1 shows the location of lumpectomy cavity (contoured in pink color as the gross tumor volume) for a right breast (a) and a left breast (b) patient included in this study. Bolus was not required due to the deep‐seated nature of the lumpectomy cavities, as shown in Fig. 1. The contralateral breast, ipsilateral lung, and heart structures were used as meaningful OARs for plan comparison.

The dose constraints to the OARs were chosen based on the NASBP B‐39/RTOG 0413^10^ in addition to those listed in RTOG 1005 study to compare standard treatments to the GsCM technique. These dose constraints were supported by the study conducted by Popescu et al.^11^ on simultaneous couch and gantry dynamic arc rotation (CG‐Darc) for APBI and Baglan et al.^12^ on accelerated partial breast irradiation using 3D‐CRT approach. Both studies found these constraints meaningful for assessing normal tissue toxicity, tumor control, and better cosmetic results.

### 2.C Treatment planning, optimization goal, and plan comparison

Eclipse treatment planning system (Varian^®^ medical systems, Palo Alto, CA) was utilized to create treatment plans based on CT simulation study [Anisotropic analytical algorithm (AAA) version 15.6; 0.2 mm dose grid resolution]. All treatment plans utilizing 6 MV photon beam were delivered on a Varian Truebeam® linear accelerator equipped with a high‐definition 120 MLC system.

#### 2.C.1 Gantry static couch motion (GsCM) technique

##### Design and feasibility test

**Fig. 2:**
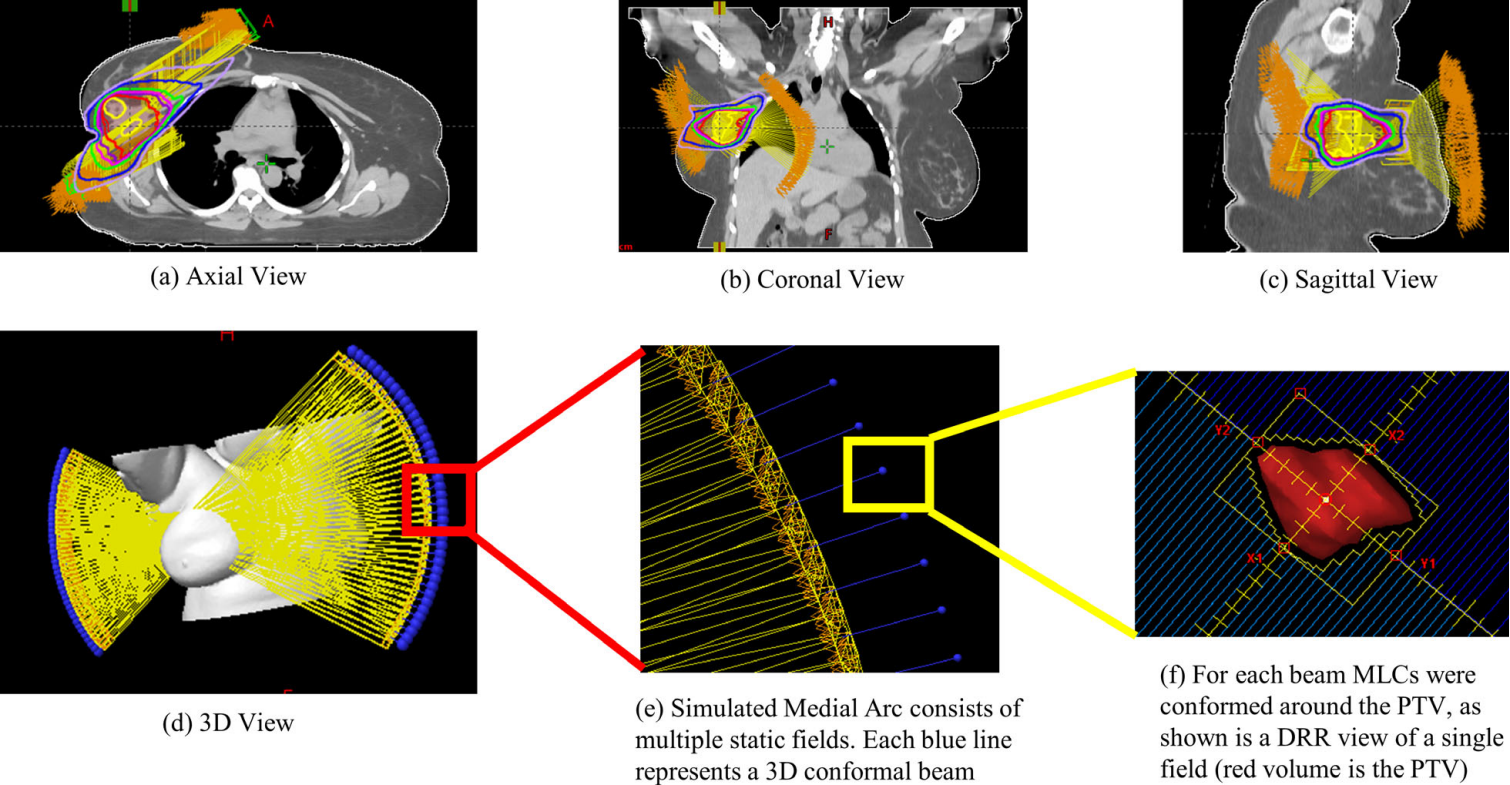
The field arrangement of gantry static couch motion (GsCM) technique for the case in Fig. 1(a). The GsCM field arrangement consists of two oblique arcs aiming at the isocenter as shown in an axial view (a), coronal view (b), sagittal view (c), and a three‐dimensional view (d). Each arc utilizes multiple static fields (e) conformed around the target volume (f). For each beam, MLCs were conformed around the planning target volume as shown in the beams‐eye‐view (f) for right‐sided target with single isocenter approach.

Twenty cases were replanned utilizing the GsCM technique. As an example, Fig. 2 shows the field arrangement of GsCM technique for the case in Fig. 1(a). The selection of the couch positions for each arc was done visually in conjunction with the 3D rendering model in the planning system which was utilized to avoid any collision of gantry with patient and couch. The start and stop couch positions were different for each patient based on their anatomy and positioning on the breast board but within ± 10° of the total couch span on each side as an average for all 20 patients.

**Fig. 3:**
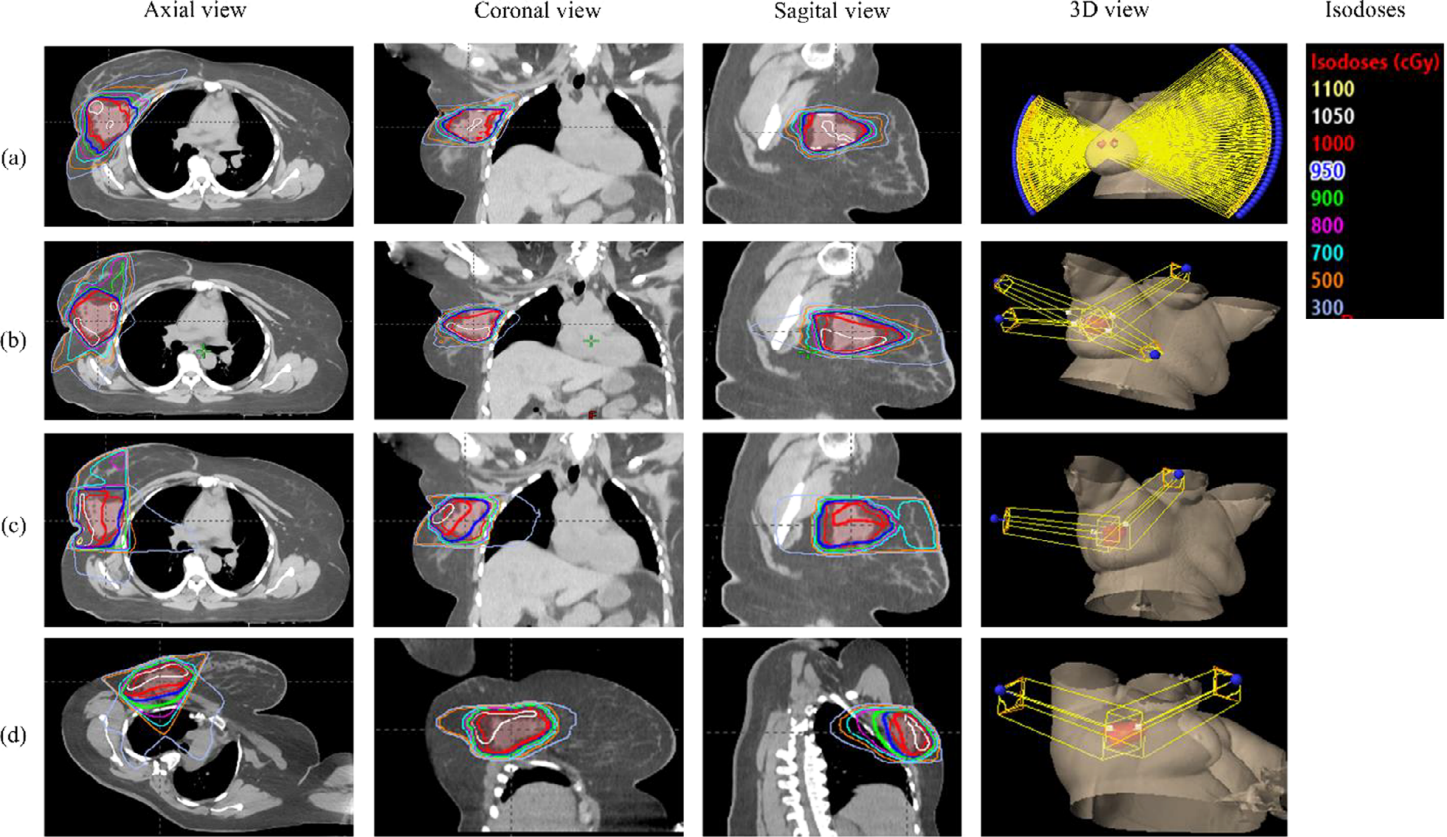
Comparison of the field arrangement and their respective axial, coronal, sagittal, and three‐dimensional (3D) view of isodoses overlay for the right breast lumpectomy boost treatment plan of the case in Fig. 1(a): (a) gantry static couch motion (GsCM), (b) 3D‐conformal radiation therapy (3D‐CRT), (c) wedge pair in supine position (WPS), and (d) wedge pair in decubitus position (WPD) techniques.

In the current clinical context, implementation of this technique is not feasible since commercially available treatment planning systems (TPS) are unable to calculate dose while the treatment couch is set in motion. Also, Truebeam® does not allow the beam ON in clinical mode during couch motion. Therefore, for the purpose of this study, simulated arcs consisting of multiple static fields were designed. Specifically, two simulated arcs were generated for the right breast case as shown in Fig. 2: (a) Medial beams with couch rotation from 40° to 320° and a fixed gantry angle at 55°, and (b) Lateral beams with couch rotation from 336° to 22° and a fixed gantry angle at 235°. Similarly, for the left breast: (a) Medial beams with couch rotation from 320° to 40° and a fixed gantry angle at 325°, and (b) Lateral beams with couch rotation from 22° to 336° and a fixed gantry angle at 145°. After creating the plan in Eclipse by simulating multiple fields for each medial and lateral arc, the treatment plan was exported to Matlab^®^ and a machine control file in XML format was generated, combining the fields into deliverable arcs. The isodose distribution for this technique is shown in Fig. 3(a).

#### 2.C.2 3D conformal external beam radiotherapy (3D‐CRT)

This technique follows the NSABP B‐39/RTOG 0413^10^ approach of choosing 3‐5 noncoplanar fields using 6 MV photons. Our 4‐field technique consists of a left anterior superior‐to‐inferior oblique (Lt ASIO), left anterior inferior‐to‐superior oblique (Lt AISO), right anterior inferior‐to‐superior oblique (Rt AISO), and right posterior superior‐to‐inferior oblique (Rt PSIO) for right breast lesions. For left breast lesions a four‐field technique consisting of right anterior superior‐to‐inferior oblique (Rt ASIO), right anterior inferior‐to‐superior oblique (Rt AISO), left posterior superior‐to‐inferior oblique (Lt PSIO), and left posterior inferior‐to‐superior oblique (Lt PISO) was used. The combination of gantry and couch positions was chosen to avoid the beam entrance and exit dose to heart and contralateral breast and minimized the exit dose to contralateral lung. The gantry angles for medial beams were deliberately steep to minimize the dose to normal breast tissue. Couch angles (20°–40°) were selected to spread out the fields and avoid collision of the gantry head and treatment couch. Each field included a 60° wedge angle and the heel of wedge was kept anteriorly for all the fields. A 5 mm margin which defines the MLC aperture around the PTV was used to account for beam penumbra. The field arrangement for this technique is shown in Fig. 3(b).

#### 2.C.3 3D planning using wedge‐pair technique in supine (WPS) and in decubitus position (WPD)

The wedge pair technique was applied to the static fields to make the dose more homogeneous within the PTV. The dose homogeneity was achieved by selecting combination of different wedge angles and hinge angles. Based on the location and depth of target, a wedge angle of 45°–60° was considered adequate for dose coverage. The fields arrangement utilizing this technique is shown in Fig. 3(c) for WPS and Fig. 3(d) for WPD.

#### 2.C.4 Plan comparison and dosimetric indices

The dose distribution for GsCM, 3D‐CRT, WPS, and WPD was compared by overlaying the isodoses on axial, coronal, and sagittal slices as shown in Fig. 3. A two‐sided paired t‐test statistical analysis was performed with *P* ≤ 0.05 considered significant. All plans were normalized equally (D95 = 100%) as recommended by ICRU report 83^13^ to compare the mean doses to PTVs. Dosimetric distributions were evaluated using the homogeneity index (H.I.),^14^ conformity index (C.I.),^15^ and gradient measure (G.M.). Briefly:

(1) $$H.I.=\left(D2\%+D98\%\right)/D50\%$$

where D98%, D2%, and D50% are dose received by 98%, 2%, and 50% of the volume. Homogeneity Index values approaching zero are considered as an ideal value for plan comparison.

(2) $$C.I.=V_{95\%}V_{\text{PTV}}$$

where V_95%_ is the volume enclosed by isodose surface of 95% prescription dose and V_PTV_ is the target volume. Conformity index approaching 1 is considered an adequate plan for comparison.

(3) $$G.M.\left(\text{cm}\right)=R_{p}+R_{50}$$

where R_p_ and R_50_ are the equivalent sphere radius of the prescription and half prescription isodoses. Gradient measure describes dose fall off from the PTV for the central slice.

### 2.D Validation of GsCM technique

**Fig. 4:**
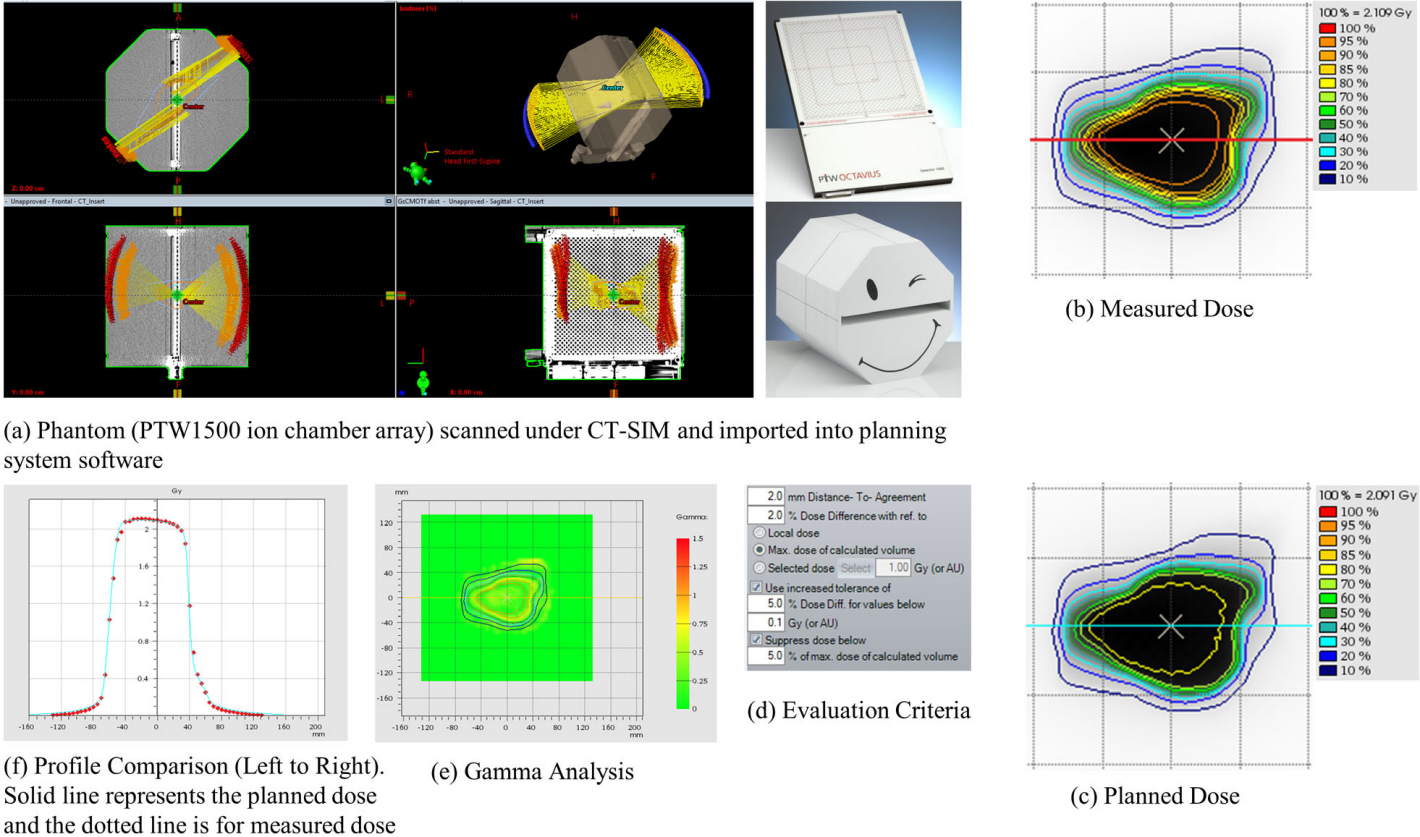
Delivery and validation of gantry static couch motion technique was conducted utilizing an ion chamber array (a) scanned in a vertical position to avoid any side beam entrance. Measured (b) and planned (c) dose were compared using a criteria (d) of 2 mm distance to agreement (DTA), 2% dose difference (%DD), and two‐dimensional gamma (e) passing rate of more and equal to 95% and dose profile comparison from left to right (transverse) direction (f).

The fields for the GsCM technique were validated utilizing IMRT QA phantom (Octavius1500, PTW‐Freiburg, Germany) as shown in Fig. 4. The QA phantom was scanned under CT simulation and imported into Eclipse planning system and the planar dose was generated for each simulated arc. The simulated arcs were delivered onto this phantom under Truebeam® developer mode application using the converted machine control file in XML format as described earlier. The measured dose was compared with the calculated dose using the criteria of percentage dose difference (%DD), distance to agreement (DTA), and 2D gamma (γ) comparison. For our study we used 2%DD, 2 mm DTA, and γ ≤ 1 with passing criteria of ≥95% which is standard of care in our institution for all IMRT patients.

## 3 RESULTS

### 3.A Group A: Right breast patients

For the 10 right breast patients planned for lumpectomy boost, the mean PTV volume was 100.3 cc (range 75.8–130.5 cc) as shown in Table 1. The mean whole breast volume was 1594.6 cc (range 998.9–2705.3 cc). The lumpectomy boosts were located in the lower inner, central, upper inner, lower outer, lower inner, and upper outer quadrant of breast. The physical depth of all these cavities was greater than 5cm. An example is shown in Fig. 1(a). The fields arrangement for GsCM, 3D‐CRT, WPS, and WPD are shown in Figs. 3(a)–3(d), respectively. The dose (mean ± SD) to the normal structures has shown in Table 2 for all four techniques (GsCM, 3D‐CRT, WPS, and WPD).

**Table 2:** Right breast dosimetric parameters show improvement in planning target volume (PTV) coverage, ipsilateral lung, and heart. GsCM is superior when compared to 3D‐CRT, WPS, and WPD. All results are shown as mean ± standard deviation (ρ≤0.05 to consider statistically significant).

Parameter (objective)	GsCM	3D‐CRT	WPS	WPD	*p‐value GsCM vs 3D‐CRT*	*p‐value GsCM vs WPS*	*p‐value GsCM vs WPD*	*p‐value 3D‐CRT vs WPS*	*p‐value 3D‐CRT vs WPD*
PTV									
CI	1.1 ± 0.4	1.2 ± 0.1	1.5 ± 0.2	1.3 ± 0.1	0.451	0.001	<0.001	<0.001	0.038
GM (cm)	2.6 ± 0.3	1.5 ± 0.1	2.0 ± 0.2	1.6 ± 0.1	<0.001	0.001	<0.001	<0.001	0.038
HI	0.08 ± 0.03	0.10 ± 0.02	0.15 ± 0.02	0.11 ± 0.02	0.096	<0.001	0.017	<0.001	0.278
V95% (>95%)	97.3 ± 0.8	98.0 ± 1.2	93.7 ± 2.5	94.5 ± 2.5	0.411	<0.001	0.003	0.001	0.006
Max. dose (%)	105 ± 2.0	105 ± 2.3	106 ± 3.1	106 ± 2.1	1	0.403	0.289	0.423	0.323
Ipsilateral breast									
V50% (<60%)^a^	21.3 ± 5.1	19.1 ± 2.3	17.9 ± 1.7	17.7 ± 3.2	0.229	0.061	0.075	0.139	0.216
V100% (<35%)^a^	2.3 ± 0.5	4.2 ± 0.5	4.9 ± 1.2	8.9 ± 2.3	<0.001	<0.001	<0.001	0.106	<0.001
Mean dose (cGy)	263.6 ± 28.6	240.2 ± 23.1	184.9 ± 19.5	211.3 ± 40.9	0.059	<0.001	0.004	<0.001	0.068
Ipsilateral lung									
V30% (<15%)^a^	1.3 ± 0.5	8.1 ± 5.3	24.6 ± 7.9	44.6 ± 11.5	<0.001	<0.001	<0.001	<0.001	<0.001
D50% (cGy)	17.3 ± 5.0	90 ± 56.3	66.7 ± 56	220.3 ± 113.9	<0.001	0.022	<0.001	0.366	0.005
D30% (cGy)	29.8 ± 9.6	169 ± 32.6	230 ± 91.6	359.8 ± 54.6	<0.001	<0.001	<0.001	0.063	<0.001
Mean dose (cGy)	37.9 ± 8.7	128 ± 43.3	152.1 ± 36.9	251.4 ± 59.8	<0.001	<0.001	<0.001	0.197	<0.001
Heart									
V5% (<5%)^a^, %	0.1 ± 0.05	1.1 ± 1.0	42.8 ± 18.8	61.2 ± 21.1	0.005	<0.001	<0.001	<0.001	<0.001
D50% (cGy)	3.6 ± 0.7	6.2 ± 2.4	57.5 ± 56.4	142.7 ± 106	0.004	0.014	0.002	0.01	<0.001
D30% (cGy)	6.2 ± 1.0	10.4 ± 3.4	150.8 ± 69.5	231.9 ± 77.7	0.002	<0.001	<0.001	<0.001	<0.001
Mean dose (cGy)	5.2 ± 1.0	10.9 ± 4.4	98.6 ± 33.2	159.3 ± 55.5	<0.001	<0.001	<0.001	<0.001	<0.001
Max. dose (cGy)	31.7 ± 11.1	170.4 ± 76.9	365.4 ± 27	380.2 ± 25.5	<0.001	<0.001	<0.001	<0.001	<0.001
Contralateral breast									
Max. dose (cGy)	8.52 ± 2.5	115.79 ± 25.6	225.53 ± 65.3	350.5 ± 55.5	<0.001	<0.001	<0.001	<0.001	<0.001

Abbreviations: 3D‐CRT, three‐dimensional conformal radiotherapy, GsCM, gantry static couch motion, WPD = wedge pair decubitus, WPS = wedge pair supine.

^a^ Constraints from NASBP B‐39/RTOG 0413 are considered as meaningful endpoints including the additional constraints.

The mean conformity index (CI) and homogeneity index (HI) of 1.1 ± 0.4 and 0.08 ± 0.03 for GsCM is comparable to 3D‐CRT (1.2 ± 0.1, 0.1 ± 0.02) and superior to WPS (1.5 ± 0.2, 0.15 ± 0.02) and WPD (1.3 ± 0.1, 0.11 ± 0.02) with ρ < 0.01. The overall dose coverage to PTV, the volume getting 95% of the dose (V95%) is comparable for GsCM and 3D‐CRT (97.5%, mean), and superior to WPS and WPD (94%, mean). The gradient measure (cm) was measured to be 2.6 ± 0.3, 1.5 ± 0.1, 2.0 ± 0.2, and 1.6 ± 0.1 for the GsCM, 3D‐CRT, WPS, and WPD, respectively.

For GsCM, the ipsilateral breast volume getting 50% of the dose (V50%) was 8–10% higher and the mean dose was 10–15% higher as compared to 3D‐CRT, WPS, and WPD. But the volume getting 100% of the dose (V100%) reduced by 40%, 53%, and 75% for GsCM compared to 3D‐CRT, WPS, and WPD, respectively.

Overall, an average dose to 30% and 50% (D30%, D50%) of the ipsilateral lung volume reduced using GsCM as compared to 3D‐CRT, WPS, and WPD by 82% (range: 65%–90%), 80% (range: 74%–85%), 70% (range: 56%–80%), respectively. For GsCM, volume getting 30% of the dose (V30%) reduced by 84%, 95%, and 97% (mean) as compared to 3D‐CRT, WPS, and WPD, respectively.

For heart, the volume getting 5% of the dose (V5%) reduced by 90%, 97%, and 99% (mean) for GsCM compared to 3D‐CRT, WPS, and WPD, respectively. A similar trend of reduction was noticed for dose getting to 30% and 50% of the volume (D30%, D50%) and maximum dose.

**Fig. 5:**
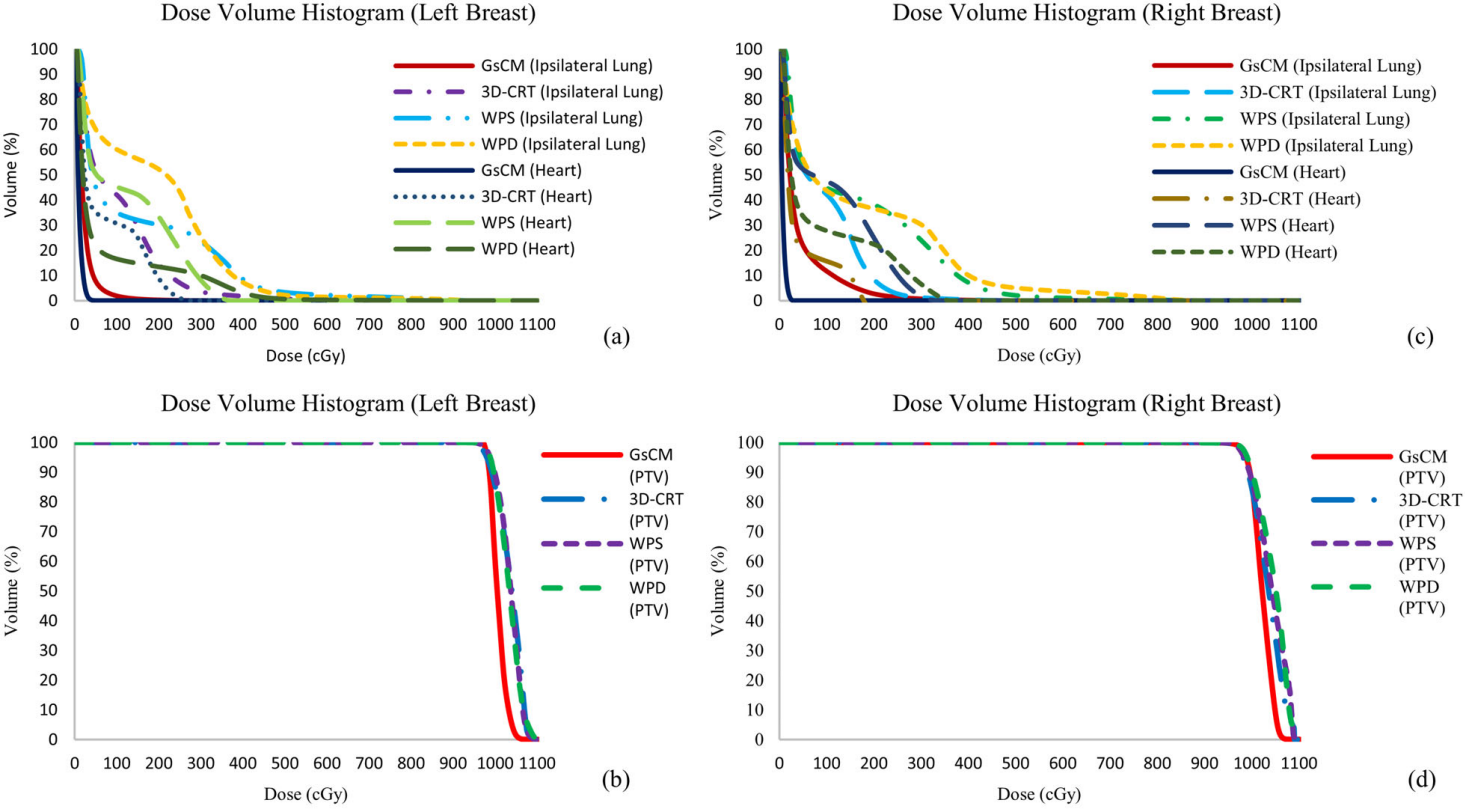
Dose volume histogram (DVH) comparison for a left breast patient (a,b) and a right breast patient (c,d). The graph shows the dose reduction to 10%, 30%, and 50% of the volume getting prescription dose to heart and ipsilateral lung using gantry static couch motion technique while maintaining same PTV coverage as compared to three‐dimensional‐conformal radiation therapy, wedge pair in supine position, and wedge pair in decubitus position.

Regardless of the location of lumpectomy cavity, the GsCM significantly lowered the contralateral breast maximum dose to under 10cGy (8.5 ± 2.5 cGy, mean ± SD, ρ ≤ 0.001) as compared to 3D‐CRT (115.8 ± 25.6 cGy, ρ ≤ 0.001), WPS (225.5 ± 65.3 cGy, ρ ≤ 0.001), and WPD (350.5 ± 55.5 cGy, ρ≤0.001), respectively, while maintaining the coverage to PTV (ρ≤0.001). The DVH comparison of a single patient with all four techniques are shown for heart, ipsilateral lung, and PTV in Figs. 5(c) and 5(d). Significant improvement in dose reduction to the ipsilateral lung and heart is noted for GsCM in terms of the dose to 30% and 50% of the volume (D30%, D50%).

The monitor units (MU) reported for GsCM, 3D‐CRT, WPS, and WPD are 390, 490, 416, and 380, respectively, for a prescription of 250 cGy per fraction for four fractions, or a total dose of 1000 cGy.

### 3.B Group B: left breast patients

For the 10 left breast patients planned for lumpectomy boost, the mean PTV volume was 105.1 cc (range 86.1–116.9 cc) as shown in Table 1. The mean whole breast volume was 1489.3 cc (range 854.5–1854.3 cc). The lumpectomy boost located in the lower inner, central, upper inner, lower outer, lower inner, and upper outer quadrant of breast. The physical depth of these cavities was greater than 5cm. A sample is shown in Fig. 1(b) for a single patient. The mean DVH comparison between all four techniques for Heart, Ipsilateral Lung, and PTV is shown in Figs. 5(a) and 5(b). The GsCM technique is considered dosimetrically superior to 3D‐CRT, WPS, and WPD as shown in Table 3.

The mean conformity index (CI) and homogeneity index (HI) of 1.0 ± 0.2, 0.08 ± 0.01, for GsCM is comparable to 3D‐CRT (1.43 ± 0.2, 0.11 ± 0.02) but superior to WPS (2.1 ± 0.2, 0.11 ± 0.01) and WPD (1.6 ± 0.2, 0.11 ± 0.01) with ρ < 0.01. The overall dose coverage to PTV, the volume getting 95% of the dose (V95%) for GsCM is comparable to 3D‐CRT (98%, mean) but superior to WPS and WPD (95%, mean). The gradient measure (cm) was measured to be 2.8 ± 0.6, 2.2 ± 0.3, 2.1 ± 0.1, and 2.0 ± 0.2 for the GsCM, 3D‐CRT, WPS, and WPD, respectively.

For GsCM, the ipsilateral breast volume getting 50% of the dose (V50%) was 15‐20% higher and the mean dose was 5–10% higher in comparison to 3D‐CRT, WPS, and WPD. But the volume getting 100% of the dose (V100%) was reduced by 32%, 63%, and 59% for GsCM compared to 3D‐CRT, WPS, and WPD, respectively.

Overall, the average dose to 30% and 50% (D30%, D50%) of the ipsilateral lung volume using GsCM was reduced by 83% (range: 79%–90%), 82% (range: 75%–85%), and 75% (range: 70%–82%) when compared to 3D‐CRT, WPS, and WPD, respectively. For GsCM, volume getting 30% of the prescription dose (V30%) was reduced by 90%, 98%, and 93% (mean) as compared to 3D‐CRT, WPS, and WPD, respectively.

For heart, the volume getting 5% of the dose (V5%) was reduced by 97%, 99%, and 98% (mean) for GsCM compared to 3D‐CRT, WPS, and WPD, respectively. Same trend of reduction was noted for dose getting to 30% and 50% of the heart volume (D30%, D50%) and the maximum dose.

Regardless of the location of lumpectomy cavity, the GsCM significantly decreased the contralateral breast maximum dose to be under 5 cGy (3.1 ± 1.1, mean ± SD, ρ ≤ 0.001) as compared to 3D‐CRT (129.7 ± 22.3, ρ ≤ 0.001), WPS (215.5 ± 68.3, ρ ≤ 0.001), and WPD (310.6 ± 47.8, ρ≤0.001), respectively, while maintaining the coverage to PTV (ρ≤0.001).

The total monitor units (MU) reported for GsCM, 3D‐CRT, WPS, and WPD were 400, 480, 410, and 385, respectively, for a prescription of 250 cGy per fraction for four fractions, corresponding to a total dose of 1000 cGy.

### 3.C Dose delivery and validation

All GsCM plans were successfully converted into a single deliverable arc utilizing the in‐house MATLAB^®^ software that combines multiple static fields into one deliverable arc in an XML format consisting of multiple segments of MLC apertures, monitor units, couch, and gantry positions. These XML files were delivered onto PTW Octavius ion chamber array to assess the accuracy of delivery. Comparison of simulated arcs and measured dose distributions utilizing GsCM yielded 98 ± 1.5% (range 96.5–100%) easily satisfying our institutional criteria. The median two‐dimensional gamma index was of 0.35 and absolute median dose difference was of 0.0256 Gy. The delivery time for simulated arcs was significantly shorter (<2 min) as compared to 3D‐CRT and comparable to wedge pair techniques (~1.5 min) at the dose rate of 600 MU/min. This includes the time to prepare each field for each technique.

## 4 DISCUSSION

In this study, the GsCM technique was dosimetrically compared with 3D‐CRT, WPS, and WPD techniques for breast boost treatments. All patients selected for this study had undergone combined whole breast radiation therapy and photon boost to deeply seated lumpectomy cavities. Instead, 3D‐CRT and WPS are typically used for treating these patients in supine position. Sometimes, decubitus position is utilized for patients who have lumpectomy cavity sitting under the breast fold to achieve adequate coverage and avoid any skin reaction from dose build up under the breast fold. The GsCM technique was considered because it provides a very comfortable alternative position (supine) to the decubitus position. Our technique is more like a VMAT type of dose delivery but no traditional inverse optimization. This is due to the inability to limit the fields to a single segment. We limited to a single segment to best mimic VMAT.

In this study, comparison of dose distribution (Conformity Index), dose fall off (Gradient Index), and dose volume histograms (V95%) of each technique revealed similarities between GsCM and 3D‐CRT and superiority of GsCM over WPS and WPD. For ipsilateral breast volume, V50% and mean dose were higher using GsCM because unlike other techniques, the tangent beams for GsCM are parallel to chest wall and do not exit through ipsilateral lung and/or heart. On the other hand, for all cases (right and left breast), the beam arrangement of GsCM significantly reduced dose to 30% and 50% of heart and ipsilateral lung volume getting prescription dose (D30% and D50%), and mean and maximum dose to heart volume (ρ≤0.01). This indicates that although GsCM confers additional conformality and OAR sparing, there is still a trade‐off with regards to low‐dose spillage to normal breast tissue. Overall, GsCM resulted in significantly (ρ≤0.01) lower dose to ipsilateral lung (V30%, D50%, D30%, and mean dose), heart volume getting V5%, D50%, D30%, and max dose, contralateral breast max dose, and resulted in better conformity and homogeneity index. The monitor units prescribed for GsCM were relatively lower than 3D‐CRT and comparable to wedge pair techniques.

Our results supported the study conducted by Shaitelman et al.,^16^ where 3D‐CRT significantly reduced ipsilateral breast V50% by the amount of 15–40% (mean). Fahimian et al.^17^ used LINAC‐based approach for trajectory‐modulated prone breast irradiation, a nonisocentric approach, showed significant improvement in conformity, less spread of dose to normal breast (V50%, V100%), parameters related to toxicity, negligible dose to ipsilateral lung, and heart structure. Our GsCM approach is focused on single‐isocenter approach for patients scanned in supine position, a position more appealing for faster dose delivery and patient comfort.

The delivery of multiple static fields at different couch positions is very time consuming and led us to merge all static fields to create a single deliverable arc. This was easily accomplished using MATLAB^®^ scripting by creating XML files. The implementation and delivery of GsCM was conducted in the Truebeam® developer mode application because simultaneous dose delivery and couch motion is not available in clinical mode. The developer mode application allowed GsCM technique to be automated and faster as compared to delivering all the fields individually. We found that using these features, GsCM can be easily implemented in clinic, once dose delivery and couch motion are allowed in clinical mode.

Implementation of the GsCM technique is limited by characterization of patient motion and mitigation of potential collision during dose delivery as well as couch and gantry motion. In order to address this risk, 3D modeling of the planning system can be utilized for gantry and couch angles selection to avoid potential collision. Furthermore, utilization of laser guard interlocks during treatment delivery can be considered. With regards to patient motion, a wireless bra/custom mesh type overlay on the patient can be used to minimize motion and ensure setup repeatability between fractions. To study the couch motion, patient motion, and couch speed integration during beam delivery are not part of this study and will be discussed in a future study.

One of the limitations uncovered in this study was the short couch angular span on the lateral side of the beam for both right and left breast patients who have lumpectomy cavity located more medially. This can be mitigated, using two separate isocenters, one for each arc so that wider angular span can be achieved for both arcs. For the medial arc, the isocenter is usually positioned at the geometric center of the boost volume since there is enough clearance for large angular spans no matter where the boost volume is located. For the lateral arc, collision can be a major concern if the boost volume is located close to the medial side of patient. For cases like this, in order to encompass the whole PTV within the treatment field of view, the couch needs to be moved anteriorly and laterally from the isocenter of the medial arc, both shifts can be performed automatically within our GsCM technique framework. The data presented in this study are for single isocenter technique only.

The reduction in high dose to normal breast tissue, spread of low dose to ipsilateral lung and heart, and significant dose conformity around the target volume makes GsCM technique suitable for breast boost applications. This approach is unique and opens the possibility for future advancement for existing treatment planning techniques where the current dose calculation and dose optimization algorithms can be modified to perform GsCM type of treatment planning. The GsCM technique also provides an opportunity to take advantage of the capabilities of LINAC and treatment planning systems for other body sites where the conformity and spread of low dose to organs at risk are highly appreciated.

## 5 CONCLUSIONS

In conclusion, we presented in this study a novel GsCM treatment technique for breast boost radiation therapy. This technique utilizes medial and lateral arcs created by automatic couch motion at larger angular span with a fixed gantry position. The dosimetric properties and novelty of the GsCM technique with patient in the supine position were compared with standard 3D‐conformal breast boost treatment planning techniques. We demonstrated that the GsCM technique provides a compact and conformal dose distribution to deep‐seated breast surgical cavities where electrons would not be applicable. Good agreement was observed between the measured and calculated dose distribution. Due to the use of tangential fields placement, the GsCM technique produced no exit dose to contralateral/ipsilateral lung and heart and therefore lead to a significant dose reduction to surrounding critical organs in comparison to other photon boost techniques. Single and/or dual isocentric‐based oblique arcs with large medial and lateral angular span are adequate for breast boost applications with a shorter delivery time of ~2 min in a more convenient treatment position for the patient.

## CONFLICT OF INTEREST

There is no conflict of interest declared in this article.

6

**Table 3:** Left breast dosimteric parameters show improvement in PTV coverage, ipsilateral lung, and heart. GsCM is superior when compared to 3D‐CRT, WPS, and WPD. All results are shown as mean ± standard deviation (ρ≤0.05 to consider statistically significant).

Parameter (objective)	GsCM	3D‐CRT	WPS	WPD	*p‐value GsCM vs 3D‐CRT*	*p‐value GsCM vs WPS*	*p‐value GsCM vs WPD*	*p‐value 3D‐CRT vs WPS*	*p‐value 3D‐CRT vs WPD*
PTV									
CI	1.0 ± 0.2	1.43 ± 0.2	2.1 ± 0.2	1.6 ± 0.2	<0.001	<0.001	<0.001	<0.001	0.074
GM (cm)	2.8 ± 0.6	2.15 ± 0.3	2.1 ± 0.1	2.0 ± 0.2	0.004	0.002	0.004	0.623	0.205
HI	0.08 ± 0.01	0.11 ± 0.02	0.12 ± 0.01	0.11 ± 0.01	<0.001	<0.001	<0.001	0.174	1
V95% (>95%)	98.1 ± 0.3	98 ± 0.25	95.4 ± 0.4	95.0 ± 0.9	<0.001	<0.001	<0.001	<0.001	<0.001
Max. dose (%)	105 ± 2.1	105 ± 2.3	105.2 ± 2.8	105.4 ± 2.5	<0.001	<0.001	<0.001	0.863	0.714
Ipsilateral breast									
V50% (<60%)^a^	14.8 ± 4.5	12.1 ± 1.9	17.5 ± 2.2	20.1 ± 4	0.097	0.106	0.012	<0.001	<0.001
V100% (<35%)^a^	1.9 ± 0.3	2.8 ± 0.6	5.1 ± 0.7	4.6 ± 0.7	<0.001	<0.001	<0.001	<0.001	<0.001
Mean dose (cGy)	171.3 ± 36.6	162.3 ± 23.9	177.2 ± 21.1	219.2 ± 28.9	0.523	0.664	0.005	0.157	<0.001
Ipsilateral lung									
V30% (<15%)^a^	1.0 ± 0.8	11 ± 4.9	29.1 ± 7.4	14.2 ± 8.8		<0.001	<0.001		0.328
D50% (cGy)	15.5 ± 2.8	89.7 ± 12.2	82.3 ± 68.4	97.2 ± 76	<0.001	0.012	0.008		0.762
D30% (cGy)	25.8 ± 5.1	163 ± 14.5	243.2 ± 117.9	194.3 ± 102.9	<0.001	<0.001	0.001	<0.001	0.354
Mean dose (cGy)	31.9 ± 9.4	126 ± 15.2	177.7 ± 37.8	137.1 ± 44.9	<0.001	<0.001	<0.001	0.742	0.469
Heart									
V5% (<40%)^a^, %	0.5 ± 0.3	20.7 ± 7	51.9 ± 21.4	30.5 ± 20.4	<0.001	<0.001	<0.001	<0.001	0.168
D50% (cGy)	6.9 ± 0.5	57 ± 5.0	99.5 ± 50	60.7 ± 35.5	<0.001	0.021	0.041	0.016	0.748
D30% (cGy)	10.9 ± 0.8	163 ± 7.5	185 ± 102	127.7 ± 100	<0.001	<0.001	0.021	0.505	0.28
Mean dose (cGy)	9.7 ± 0.5	55.1 ± 5.0	143 ± 51.9	96.7 ± 61	<0.001	<0.001	0.001	<0.001	0.046
Max. dose (cGy)	100.1 ± 53.7	503.3 ± 15.6	647.6 ± 241.2	597 ± 184.4	<0.001	<0.001	<0.001	0.075	0.127
Contralateral breast									
Max. dose (cGy)	3.1 ± 1.1	129.7 ± 22.3	215.5 ± 68.3	310.6 ± 47.8	<0.001	<0.001	<0.001	0.001	<0.001

Abbreviations: 3D‐CRT, three‐dimensional conformal radiotherapy; GsCM, gantry static couch motion; WPD, wedge pair decubitus; WPS, wedge pair supine.

^a^ Constraints from NASBP B‐39/RTOG 0413 are considered as meaningful endpoints including the additional constraints.
